# Regulation of UVR8 photoreceptor dimer/monomer photo‐equilibrium in Arabidopsis plants grown under photoperiodic conditions

**DOI:** 10.1111/pce.12724

**Published:** 2016-05-06

**Authors:** Kirsten M.W. Findlay, Gareth I. Jenkins

**Affiliations:** ^1^ Institute of Molecular, Cell and Systems BiologyCollege of Medical, Veterinary and Life Sciences, Bower BuildingUniversity of GlasgowGlasgowG12 8QQUK

**Keywords:** photoreceptor, RUP proteins, UV‐B, UVR8

## Abstract

The UV RESISTANCE LOCUS 8 (UVR8) photoreceptor specifically mediates photomorphogenic responses to UV‐B. Photoreception induces dissociation of dimeric UVR8 into monomers to initiate responses. However, the regulation of dimer/monomer status in plants growing under photoperiodic conditions has not been examined. Here we show that UVR8 establishes a dimer/monomer photo‐equilibrium in plants growing in diurnal photoperiods in both controlled environments and natural daylight. The photo‐equilibrium is determined by the relative rates of photoreception and dark‐reversion to the dimer. Experiments with mutants in REPRESSOR OF UV‐B PHOTOMORPHOGENESIS 1 (RUP1) and RUP2 show that these proteins are crucial in regulating the photo‐equilibrium because they promote reversion to the dimer. In plants growing in daylight, the UVR8 photo‐equilibrium is most strongly correlated with low ambient fluence rates of UV‐B (up to 1.5 *μ*mol m^−2^ s^−1^), rather than higher fluence rates or the amount of photosynthetically active radiation. In addition, the rate of reversion of monomer to dimer is reduced at lower temperatures, promoting an increase in the relative level of monomer at approximately 8–10 °C. Thus, UVR8 does not behave like a simple UV‐B switch under photoperiodic growth conditions but establishes a dimer/monomer photo‐equilibrium that is regulated by UV‐B and also influenced by temperature.

## Abstract 2

UV‐B absorption by the photoreceptor UVR8 induces dimer dissociation, forming monomers that initiate photomorphogenic UV‐B responses. However, the regulation of dimer/monomer status in plants growing under photoperiodic conditions has not been examined. Here we show that UVR8 does not behave like a simple UV‐B switch under photoperiodic conditions in both controlled environments and natural daylight, but establishes a dimer/monomer photo‐equilibrium that is regulated by UV‐B and influenced by temperature.

## Introduction

Ultraviolet‐B radiation (UV‐B; 280–315 nm) in sunlight has the potential to damage organisms, although plants growing in natural conditions rarely show signs of UV‐stress because they have evolved effective systems for UV‐protection and damage repair. Exposure to low doses of UV‐B stimulates gene expression responses that enable plants to acclimate to UV‐B and hence tolerate subsequent exposure to high levels of UV‐B (Frohnmeyer & Staiger 2003; Ulm & Nagy 2005; Jenkins 2009). Moreover, UV‐B wavelengths in sunlight are important in regulating a wide range of plant processes, including metabolic activities, morphogenesis, photosynthetic competence and defence against pests and pathogens (Jordan 1996; Jenkins 2009; Ballaré *et al*. 2012; Robson *et al*. 2014). It is therefore important to understand the cellular and molecular mechanisms that enable plants to detect and respond to UV‐B.

UV‐B exposure stimulates the differential expression of hundreds of plant genes (Casati & Walbot 2004; Ulm *et al*. 2004; Brown *et al*. 2005; Kilian *et al*. 2007; Favory *et al*. 2009). In many cases these responses are initiated by activation of non‐UV‐B‐specific signalling pathways, involving DNA damage or increases in the levels of reactive oxygen species or defence or wound signalling molecules (Kilian *et al*. 2007; Jenkins 2009; Gonzalez Besteiro *et al*. 2011). However, many genes are regulated through activation of the UV‐B photoreceptor UV RESISTANCE LOCUS 8 (UVR8) (Brown *et al*. 2005; Favory *et al*. 2009). UVR8 is highly conserved in the plant kingdom and mediates a variety of responses to UV‐B, including UV‐protective sunscreen biosynthesis, suppression of hypocotyl extension, leaf expansion, phototropism, stomatal closure and inhibition of the shade avoidance response (Wargent *et al*. 2009; Tilbrook *et al*. 2013; Hayes *et al*. 2014; Jenkins 2014; Tossi *et al*. 2014; Vandenbussche *et al*. 2014).

UVR8 is a seven‐bladed β‐propeller protein that exists as a dimer in the absence of UV‐B (Rizzini *et al*. 2011; Christie *et al*. 2012; Wu *et al*. 2012). The dimer is formed by electrostatic interactions between charged amino acids at the surface where monomers come into contact (Christie *et al*. 2012; Wu *et al*. 2012). Unlike other photoreceptors, UVR8 does not employ an attached cofactor to absorb light of particular wavelengths, and instead uses specific tryptophan amino acids in its primary sequence for UV‐B photoreception (Rizzini *et al*. 2011; Christie *et al*. 2012; O'Hara & Jenkins 2012; Wu *et al*. 2012; Huang *et al*. 2014; Zeng *et al*. 2015). Photoreception causes dissociation of the dimer, enabling monomeric UVR8 to initiate signal transduction by interaction with the CONSTITUTIVELY PHOTOMORPHOGENIC 1 (COP1) protein (Rizzini *et al*. 2011). UVR8 and COP1 together activate processes that regulate transcription of target genes involved in UV‐B responses (Favory *et al*. 2009). Following photoreception, UVR8 monomers are able to re‐associate to form dimers (Heijde & Ulm 2013; Heilmann & Jenkins 2013). This process is facilitated *in vivo* by other proteins, in particular REPRESSOR OF UV‐B PHOTOMORPHOGENESIS 1 (RUP1) and RUP2 (Heijde & Ulm 2013). Because the RUP proteins promote re‐dimerization, they constrain responses initiated by the UVR8 monomer; hence *Arabidopsis rup1*,*rup2* mutant plants exhibit enhanced responses to UV‐B mediated by UVR8 (Gruber *et al*. 2010). Expression of the *RUP* genes is stimulated by UV‐B, mediated by UVR8 and COP1, and hence provides a negative feedback regulation of UVR8 activity (Gruber *et al*. 2010).

Our present understanding of UVR8 photoreceptor function is derived principally from studies with either the purified protein or plants grown and treated under laboratory conditions. For instance, in vivo studies of UV‐B induced UVR8 monomerization have used seedlings or young plants grown under continuous illumination with white light lacking UV‐B, which are then given acute exposure to UV‐B and monitored over relatively short time courses (Rizzini *et al*. 2011; O'Hara & Jenkins 2012; Heijde & Ulm 2013; Heilmann & Jenkins 2013). Under these conditions, the dimer is rapidly and substantially converted to the monomer. However, it is important to study the dimer/monomer status of UVR8 over longer term, low‐dose UV‐B exposure because, firstly, monomers have the potential to re‐dimerize, secondly, expression of the RUP proteins, which promote re‐dimerization, is stimulated by UV‐B treatment and, thirdly, plants often experience such conditions in the natural environment. Here we have monitored the dimer/monomer status of UVR8 in plants growing in diurnal photoperiods, both in controlled environment chambers with supplementary UV‐B, and in natural sunlight. The experiments show that UVR8 does not simply switch from dimer to monomer following UV‐B treatment, but forms a photo‐equilibrium under longer‐term UV‐B exposure that is dependent on the relative rates of monomerization and reversion to the dimer. We further show that RUP proteins play a key role in regulating the UVR8 photo‐equilibrium.

## Materials and Methods

### Plant materials and treatments

Seeds of wild‐type *Arabidopsis thaliana* ecotype Landsberg *erecta* (L*er*) and Columbia (Col‐0) were obtained from the Nottingham Arabidopsis Stock Centre (NASC). The *rup1*,*rup2* double mutant (Col‐0 background) was produced by crossing the single mutants *rup1‐1* and *rup2‐1*, obtained from NASC.

For experiments in controlled environment conditions, seeds were sown on compost, vernalized at 4 °C for 48 h, and then grown at 20 °C in a 12 h dark/12 h white light photoperiod (120 *μ*mol m^−2^ s^−1^; warm white fluorescent tubes, Osram) with either no UV‐B, or supplementary UV‐B at one of 3 fluence rates, as indicated in the figure legends. UV‐B was provided by narrowband UV‐B tubes (Philips TL20W/01RS; spectrum shown in Cloix *et al*. 2012). Plants were grown for 21 days under these conditions prior to the preparation of whole cell protein extracts. Total leaf area was measured using ImageJ software.

For experiments in daylight, seeds sown and vernalized as above were transferred to a controlled environment room with constant 120 *μ*mol m^−2^ s^−1^ white light for 7 days. Plants were then grown outside in Glasgow, UK, for a further 14 days before being sampled to prepare whole cell extracts on the dates and times indicated in the figures. Where stated, plants were shielded from UV‐B using a polyester filter (Autostat CT5, MacDermid Autotype).

To monitor UVR8 monomerization in response to UV‐B (Fig. 2a), plants were grown on agar plates containing half‐strength Murashige and Skoog (MS) salts under 120 *μ*mol m^−2^ s^−1^ constant white light (warm white fluorescent tubes, Osram) at 20 °C for 10 d. Plants were placed in darkness for 16 h before being exposed to 3 *μ*mol m^−2^ s^−1^ broadband UV‐B (Q‐panel UV‐B‐313 tubes; spectrum shown in Cloix *et al*. 2012). Samples were harvested over a time course as indicated in Fig. 2a.

For assays of reversion from monomer to dimer, plants grown on agar plates as above were transferred to low fluence rate white light (20 *μ*mol m^−2^ s^−1^) for 24 h at the selected experimental temperature (indicated in Fig. 8), before being exposed to 21 *μ*mol m^−2^ s^−1^ broadband UV‐B for 15 min to induce UVR8 monomerization. Plants were then transferred to darkness for the durations shown in Fig. 8 before being harvested to make whole cell extracts. Plants were kept at the selected treatment temperature throughout UV‐B exposure and subsequent darkness.

UV‐B fluence rates were measured using a Spectrosense 1 meter (Skye Instruments) fitted with a SKU 430 sensor, and PAR was measured with a Li‐Cor LI‐250A meter.

### UVR8 protein analysis

Arabidopsis whole cell extracts were prepared from leaf samples as described previously (Kaiserli & Jenkins 2007). The dimer/monomer status of UVR8 in whole cell extracts was determined using the method of Rizzini *et al*. (2011) as described previously (Cloix *et al*. 2012; O'Hara & Jenkins 2012; Heilmann & Jenkins 2013). To each sample, 4x SDS sample buffer (250 mM Tris–HCl pH 6.8, 2% SDS, 20% β‐mercaptoethanol, 40% glycerol, 0.5% bromophenol blue) was added, and the protein samples were loaded on a 10% SDS‐PAGE gel without boiling (unless stated otherwise). Following electrophoresis and western blotting, the immunoblots were incubated with an anti‐UVR8 antibody directed against the C‐terminus of the protein (Kaiserli & Jenkins 2007). In addition, the immunoblots were stained with Ponceau S to reveal the Rubisco large subunit (rbcL), which was used as a loading control. The data shown are representative of at least three independent experiments.

Quantification of UVR8 dimer and monomer was undertaken for all immunoblots of non‐boiled samples. The immunodetected dimer and monomer bands were visualized by chemiluminescent imaging using the Fusion FX7 instrument (peqlab, UK) and quantified using Image J software. Data were corrected for background, and the value for the dimer was normalized against values for the total amount of UVR8 (dimer and monomer), taken as 100%, to give %[UVR8^dimer^/UVR8^total^]. For assays of reversion of the monomer to the dimer in darkness, data were normalized against the total amount of UVR8 dimer prior to UV‐B exposure.

### Statistical analysis

All statistical analysis was done using R. Generalized linear models were fitted. Log‐likelihood ratio tests and Akaike Information Criterion tests were used to determine the best fitting model. ANOVA was used to determine statistical significance of different factors regulating UVR8 photo‐equilibrium.

### Accession numbers

The Arabidopsis Genome Initiative locus identifier for UVR8 is At5g63860, for RUP1 is At5g52250 and for RUP2 is At5g23730.

## Results

### UVR8 forms a dimer/monomer photo‐equilibrium in plants grown in light/dark cycles with supplementary UV‐B

In the present study *Arabidopsis* plants were grown for 3 weeks in a light/dark cycle under white light supplemented with narrowband UV‐B (λ_max_ 311 nm) at different fluence rates. The highest fluence rate used (3 *μ*mol m^−2^ s^−1^) is approximately 65% of the maximum level of UV‐B in summer sunlight in Glasgow, UK. Consistent with previous reports (Hectors *et al*. 2007; Wargent *et al*. 2009) the presence of UV‐B affected plant morphology; supplementary UV‐B stimulated a small increase in leaf area at the lowest fluence rate, and reduced leaf area at higher fluence rates (Fig. S1).

The total amount of UVR8 in leaf protein extracts, along with the relative amounts of UVR8 dimer and monomer, was measured on western blots by immunodetection with an anti‐UVR8 antibody, followed by quantification of band intensities (Cloix *et al*. 2012; O'Hara & Jenkins 2012; Heilmann & Jenkins 2013). An example of the quantification is shown in Fig. S2. The total amount of UVR8 is determined relative to the control rbcL protein and the dimer/monomer status is expressed as %[UVR8^dimer^/UVR8^total^].

**Figure 1:**
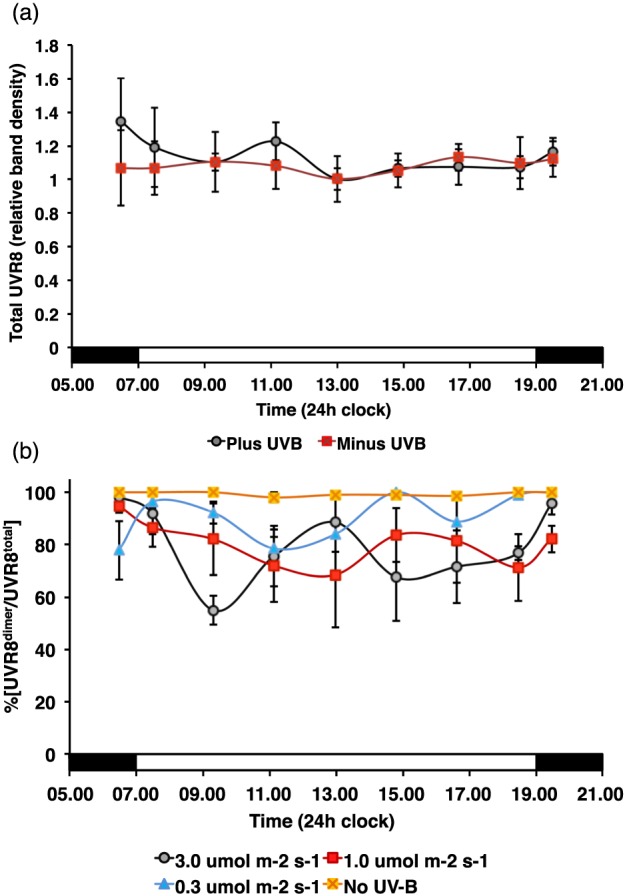
Total amount and photo‐equilibrium of UVR8 in Col‐0 plants grown in controlled environment conditions with supplementary UV‐B. Plants were grown for 3 weeks in 12 h dark/12 h 120 *μ*mol m^−2^ s^−1^ white light with either no UV‐B or supplementary UV‐B at 0.1, 1.0 or 3.0 *μ*mol m^−2^ s^−1^. Samples were harvested during the light period at the times indicated and 30 min before the start, and 30 min after the end of the light period. (a) Total amount of UVR8 in leaf samples, normalized to the rbcL loading control, measured for plants either not exposed to UV‐B or given supplementary UV‐B (3.0 *μ*mol m^−2^ s^−1^); data are the mean + S.E. (*n* = 3). (b) UVR8 photo‐equilibrium (%[UVR8^dimer^/UVR8^total^]) measured for plants grown as above. Data are the mean + S.E. (*n* = 3).

Little change was observed in the total amount of UVR8 over the diurnal period, regardless of the presence of UV‐B (Fig. 1a), consistent with previous reports that UVR8 shows largely constitutive expression (Kliebenstein *et al*. 2002; Kaiserli & Jenkins 2007). The %[UVR8^dimer^/UVR8^total^] (Fig. 1b) was very high (>95%) throughout the photoperiod in plants not exposed to supplementary UV‐B. In plants exposed to UV‐B, the %[UVR8^dimer^/UVR8^total^] was lower, but did not drop below 50%, and the mean was approximately 75%. In three experiments, the %[UVR8^dimer^/UVR8^total^] observed in each of the plus‐UV‐B treatments was significantly different to that seen in minus UV‐B (*p* < 0.05 for 0.3 *μ*mol m^−2^ s^−1^, and *p* < 0.001 for the other fluence rates). In addition, there was some evidence of a trend of increased monomerization with increased UV‐B fluence rate; %[UVR8^dimer^/UVR8^total^] at 0.3 *μ*mol m^−2^ s^−1^ was significantly different to that at the other fluence rates (*p* < 0.05 for 1.0 *μ*mol m^−2^ s^−1^, and *p* < 0.001 for 3.0 *μ*mol m^−2^ s^−1^), but %[UVR8^dimer^/UVR8^total^] did not differ significantly between 1.0 and 3.0 *μ*mol m^−2^ s^−1^ (*p* > 0.05). However, there was no significant change in %[UVR8^dimer^/UVR8^total^] with time during the photoperiod (*p* > 0.05). Similar data were observed for both Col‐0 and L*er* ecotypes (Fig. 1b; Fig. S3).

The above data show that UVR8 does not simply switch from dimer to monomer when plants are exposed to UV‐B at the start of the photoperiod. Instead, UVR8 rapidly forms a dimer/monomer photo‐equilibrium with approximately 75% dimer under the conditions used, with some change with respect to the UV‐B fluence rate, but no consistent change with time during the photoperiod.

### UVR8 photo‐equilibrium is regulated by RUP proteins

The UVR8 photo‐equilibrium (the balance in relative amounts of UVR8 dimer and monomer under illumination) can be represented by the following equation:

$$UVR8^{\text{dimer}}\overset{K_{p}}{\underset{K_{r}}{⇄}}\;UVR8^{\text{monomer}}$$

where *K_p_* is the rate constant for photoreception and *K_r_* is the rate constant for reversion of monomers to dimer.

The rate of monomerization (*K_p_*) is dependent on UV‐B photoreception by the dimer, and will be determined by the effective dose of UV‐B. Thus *K_p_* will be dependent on the wavelength of UV‐B, relative to the absorption spectrum of UVR8, and the fluence rate and duration of exposure, which show reciprocity for a simple UVR8‐mediated response (Brown *et al*. 2009). The dependence of UVR8 monomerization on the dose of UV‐B has been demonstrated both in vitro with the purified protein (Christie *et al*. 2012; Wu *et al*. 2012) and with plant extracts (Rizzini *et al*. 2011).

The rate of reversion from the monomeric to the dimeric form (*K_r_*) is not light dependent and is stimulated by proteins that interact with UVR8 (Heijde & Ulm 2013; Heilmann & Jenkins 2013). In particular, RUP proteins promote reversion of monomers to the dimer (Heijde & Ulm 2013). Following UV‐B exposure of non‐acclimated plants, reversion under minus‐UV‐B conditions is substantially complete within approximately 1 to 2 h (Heijde & Ulm 2013; Heilmann & Jenkins 2013).

**Figure 2:**
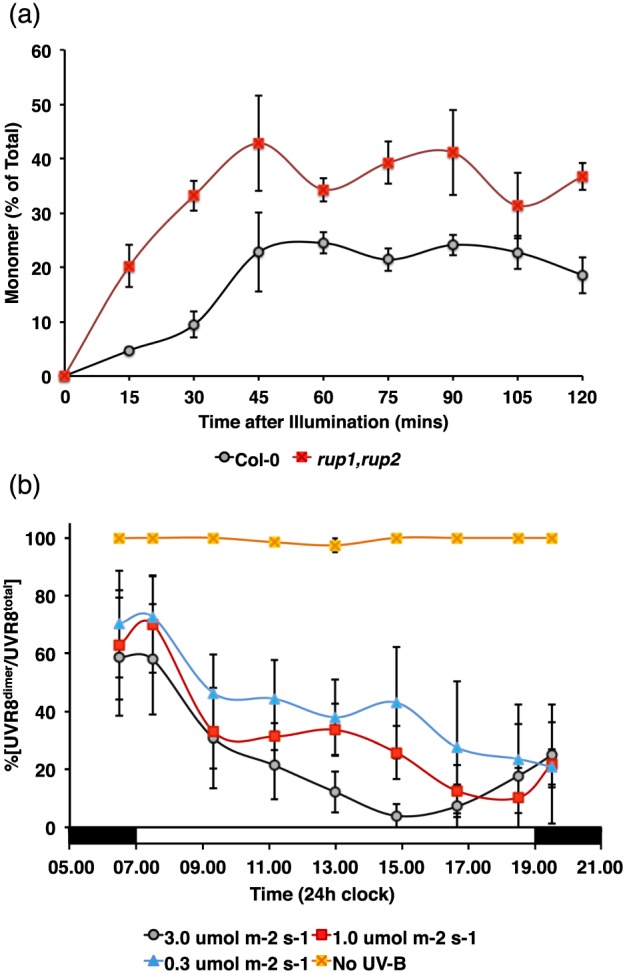
RUP proteins regulate UVR8 photo‐equilibrium. (a) Col‐0 and *rup1*,*rup2* plants were grown for 10 days in 120 *μ*mol m^−2^ s^−1^ white light, placed in darkness for 16 h, and then exposed to 3 *μ*mol m^−2^ s^−1^ UV‐B for the times indicated. The % of UVR8 in monomeric form was measured in leaf samples. (b) UVR8 photo‐equilibrium (%[UVR8^dimer^/UVR8^total^]) was measured in *rup1*,*rup2* plants grown in white light with supplementary UV‐B exactly as in Fig. 1.

According to the above equation, under constant exposure to UV‐B a photo‐equilibrium will be established dependent on the relative rates of monomerization and reversion to the dimer. In the experiments shown in Fig. 1b, *K_r_* appears to be greater than *K_p_* because the %[UVR8^dimer^/UVR8^total^] is approximately 75%. When plants not previously exposed to UV‐B were exposed to the highest UV‐B fluence rate used in the present study, partial monomerization occurred, resulting in approximately 25% monomer after 1 to 2 h (Fig. 2a). In *rup1*,*rup2* mutant plants approximately 40% monomer was observed, indicating that over the time course employed, monomerization is accompanied by reversion and that reversion is impaired in the mutant plants. In plants grown in a light/dark cycle with supplementary UV‐B, *rup1*,*rup2* plants established a photo‐equilibrium with a much lower %[UVR8^dimer^/UVR8^total^] than the wild‐type control (approximately 20%) (Fig. 2b). Thus, it is evident that RUP proteins are key regulators of the UVR8 photo‐equilibrium in plants given extended UV‐B exposure during the photoperiod.

### UVR8 photo‐equilibrium is not simply dependent on the fluence rate of UV‐B in natural daylight and is influenced by ambient temperature

The total amount of UVR8 and the UVR8 photo‐equilibrium were monitored in plants growing in natural daylight. The measurements were undertaken on 26 separate days throughout the year in Glasgow, UK, where there is substantial variation in the fluence rate of UV‐B as well as other environmental parameters, including the length of the photoperiod, temperature and the amount of photosynthetically active radiation (PAR). Plants were initially grown in a controlled environment chamber and transferred to daylight for 2 weeks prior to measurements being taken. Leaf samples were harvested to measure the total amount of UVR8 and the UVR8 photo‐equilibrium at nine points during the diurnal period, and the UV‐B fluence rate, PAR and temperature were recorded at these times.

**Figure 3:**
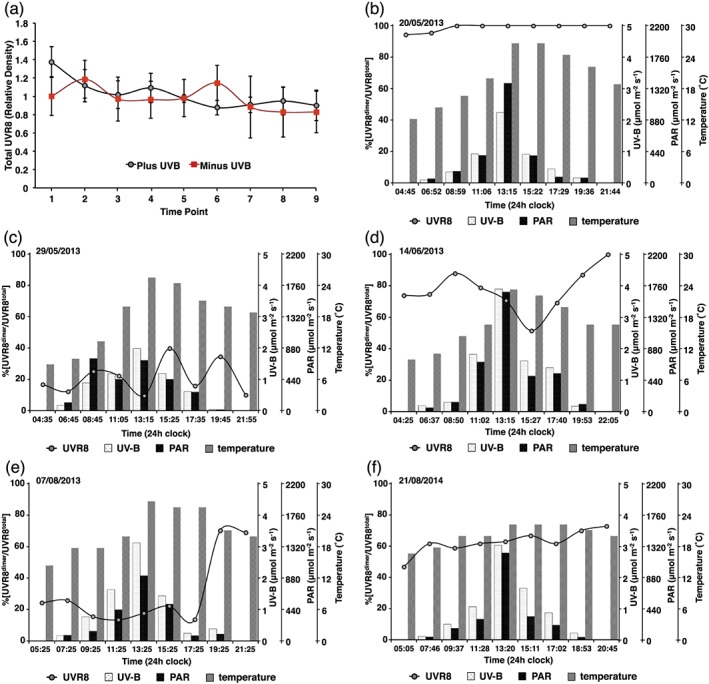
Total amount and photo‐equilibrium of UVR8 in plants grown in daylight. Plants grown for 7 days in a controlled environment were transferred to daylight for 2 weeks at various times of the year. Measurements were then made throughout a single day. The first and last time points are approximately 30 min before dawn and after dusk, respectively. The fifth time point is at solar noon. During a given day, time points are equidistant, but the length between points varies with daylength. (a) Total amount of UVR8 in leaf protein samples, normalized to the rbcL loading control, measured for plants on 6 days (in the months of January, February and May); data are the mean + S.E. Some plants were shielded from UV‐B when transferred to daylight (Minus UV‐B); others were unshielded (Plus UV‐B). (b)–(f) Measurements of UVR8 photo‐equilibrium (%[UVR8^dimer^/UVR8^total^]) made on the dates and times indicated; UV‐B, PAR and temperature were recorded at each time point.

As found under controlled environment conditions, the total amount of UVR8 showed little change during the photoperiod in plants grown in daylight (Fig. 3a). Very similar results were obtained for plants that were shielded from UV‐B, indicating that the presence or absence of UV‐B did not influence the amount of UVR8.

**Figure 4:**
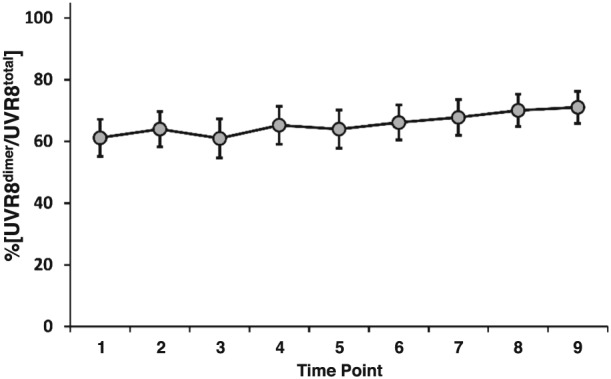
Average diurnal UVR8 photo‐equilibrium in plants grown in daylight. Plants grown for 7 days in a controlled environment were transferred to daylight for 2 weeks at various times of the year. Measurements were then made of %[UVR8^dimer^/UVR8^total^] at nine time points throughout the day. The first and last time points were approximately 30 min before dawn and after dusk, respectively. The fifth time point was at solar noon. During a given day, time points were equidistant, but the length between points varies with daylength. Data from 26 separate diurnal time courses (see Fig. S4) were combined to plot %[UVR8^dimer^/UVR8^total^] against time of day. S.E. is calculated for each time point (*n* = 26).

**Figure 5:**
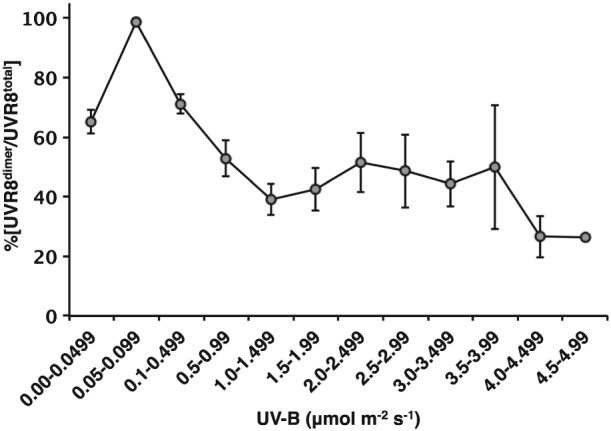
Correlation between UVR8 photo‐equilibrium and UV‐B fluence rate in plants grown in daylight. A total of 234 data points (nine time points on each of 26 days) were used to plot %[UVR8^dimer^/UVR8^total^] against UV‐B fluence rate, with fluence rates grouped as shown. S.E. is calculated for the number of data points within each UV‐B range.

A selection of the UVR8 photo‐equilibrium data is shown in Fig. 3b–f (the whole dataset is shown in Fig. S4). The UVR8 photo‐equilibrium was very variable, much more so than observed in controlled environment chambers (cf. Fig. 1b). Often, the %[UVR8^dimer^/UVR8^total^] did not appear to show a close relationship with the fluence rate of UV‐B (e.g. Fig. 3b and 3c). Furthermore, quite different %[UVR8^dimer^/UVR8^total^] could be seen on days with apparently similar environmental conditions (e.g. compare Fig. 3d, 3e & 3f). Nevertheless, when all the data from the 26 days are combined, diurnal fluctuations are cancelled out and several trends emerge. Notably, the %[UVR8^dimer^/UVR8^total^] is remarkably constant throughout the day, averaging approximately 65% (Fig. 4). In addition, generalized linear modelling analysis shows that there is a strong, statistically significant positive correlation (*p* < 0.001) between %[UVR8^dimer^/UVR8^total^] and UV‐B fluence rate. The greatest effect of UV‐B on %[UVR8^dimer^/UVR8^total^] is over the lower range of fluence rates (0.05 to 1.5 *μ*mol m^−2^ s^−1^; Fig. 5). Above 1.5 *μ*mol m^−2^ s^−1^ there is little change in %[UVR8^dimer^/UVR8^total^], although it should be noted that the sample sizes are small at the highest fluence rates.

**Figure 6:**
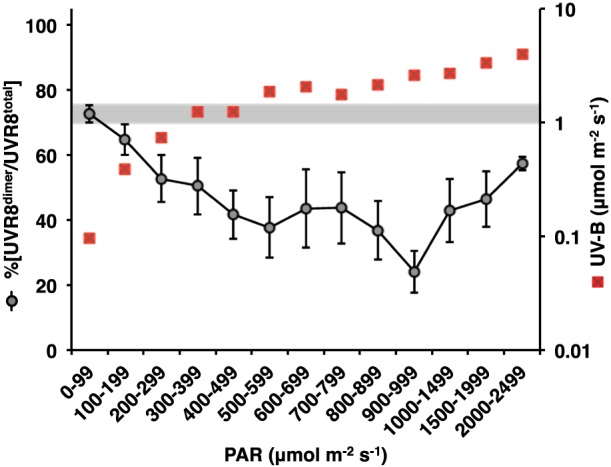
Correlation between UVR8 photo‐equilibrium and PAR in plants grown in daylight. A total of 234 data points (nine time points on each of 26 days) were used to plot %[UVR8^dimer^/UVR8^total^] against PAR, with fluence rates grouped as shown. S.E. is calculated for the number of data points within each PAR range. The mean UV‐B fluence rate is shown (red squares) for each group of PAR values. The grey bar shows the UV‐B fluence rate range (1.0 – 1.499 *μ*mol m^−2^ s^−1^) below which there is a strong correlation with %[UVR8^dimer^/UVR8^total^] (Fig. 5).

There is no evidence of a causal relationship between %[UVR8^dimer^/UVR8^total^] and PAR. UV‐B increases with PAR, as shown in Fig. 6, and it is therefore not surprising that there is a statistically significant correlation between %[UVR8^dimer^/UVR8^total^] and PAR (*p* < 0.01). However, the greatest change in %[UVR8^dimer^/UVR8^total^] with respect to PAR is over the low fluence range, where there is the strongest correlation with UV‐B (Fig. 5). The apparent increase in %[UVR8^dimer^/UVR8^total^] at high PAR is not significant and the sample size at these values is very small.

**Figure 7:**
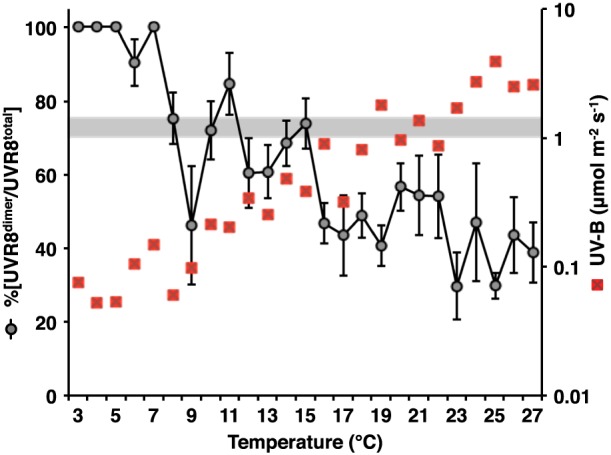
Correlation between UVR8 photo‐equilibrium and ambient temperature in plants grown in daylight. A total of 234 data points (nine time points on each of 26 days) were used to plot %[UVR8^dimer^/UVR8^total^] against temperature, with values grouped as shown. S.E. is calculated for the number of data points within each temperature range. The mean UV‐B fluence rate is shown (red squares) for each group of temperature values. The grey bar shows the UV‐B fluence rate range (1.0 – 1.499 *μ*mol m^−2^ s^−1^) below which there is a strong correlation with %[UVR8^dimer^/UVR8^total^] (Fig. 5).

A significant correlation (*p* < 0.001) was observed between ambient temperature and the UVR8 photo‐equilibrium, with %[UVR8^dimer^/UVR8^total^] decreasing with the increase in temperatures (Fig. 7). However, ambient temperature was strongly correlated with the levels of UV‐B, and it is very likely that increased monomerization up to approximately 18 °C was because of increased fluence rates of UV‐B. Above 18 °C there was little change in %[UVR8^dimer^/UVR8^total^], consistent with the lack of effect of higher UV‐B fluence rates on %[UVR8^dimer^/UVR8^total^] (Fig. 5).

**Figure 8:**
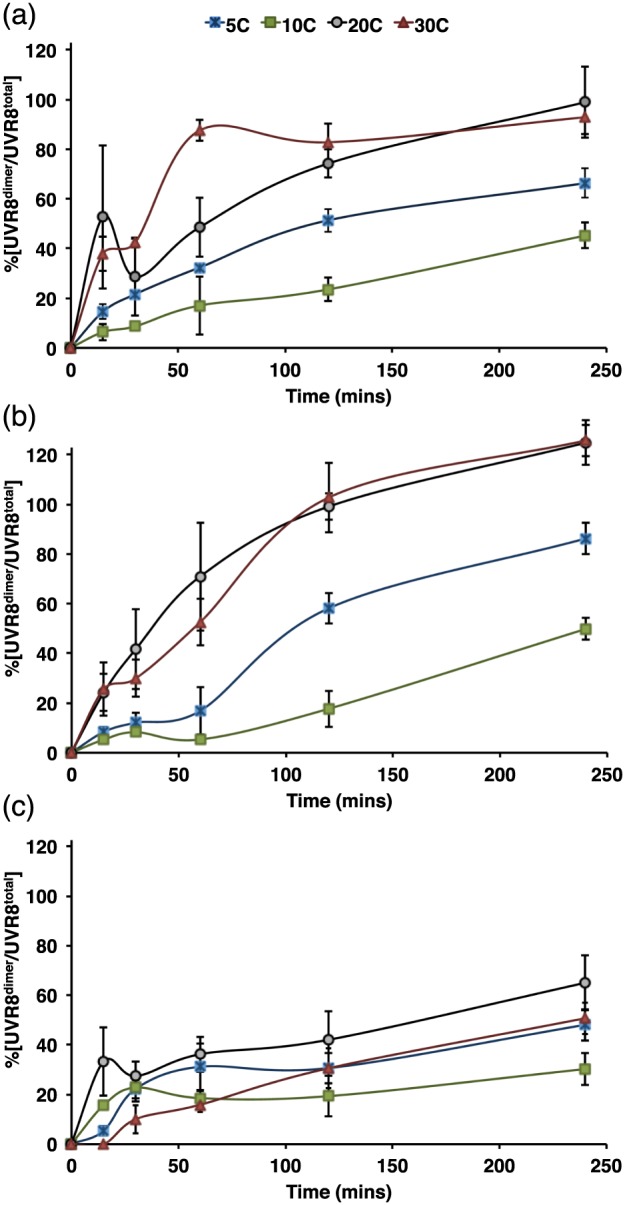
Effect of temperature on reversion of UVR8 monomer to dimer. Plants grown for 10 days in 120 *μ*mol m^−2^ s^−1^ white light were transferred to 20 *μ*mol m^−2^ s^−1^ white light for 24 h at the selected temperature and exposed to 21 *μ*mol m^−2^ s^−1^ UV‐B for 15 min to induce total UVR8 monomerization (*t* = 0). Plants were then transferred to darkness at the selected temperature for the durations indicated. The %[UVR8^dimer^/UVR8^total^] was measured in leaf samples, and is expressed relative to the total amount of dimer before UV‐B exposure (=100%). (a) L*er*. (b) Col‐0. (c) *rup1*,*rup2*.

It is interesting that a significant decrease in %[UVR8^dimer^/UVR8^total^] was observed at 8–10 °C without a corresponding step‐change in UV‐B (Fig. 7). We therefore investigated whether temperature *per se* might influence the photo‐equilibrium through the rate of reversion of monomer to dimer. In controlled environment conditions, we monitored reversion at different temperatures in plants exposed to UV‐B and then transferred to darkness. In both Col‐0 and L*er*, reversion was more rapid at higher temperatures (20 and 30 °C) than at lower temperatures, with slower reversion at 10 °C than 5 °C (Fig. 8a, b). Hence the decrease in %[UVR8^dimer^/UVR8^total^] at 10 °C in daylight‐grown plants could be explained by a reduced rate of reversion from monomer to dimer at this temperature. In *rup1*,*rup2* plants (Fig. 8c), reversion was impaired at all temperatures, but the least difference compared to the Col‐0 control was at 10 °C. These observations suggest that reversion is promoted at higher temperatures in a RUP‐dependent manner.

## Discussion

The present study extends understanding of UVR8 photoreceptor function by providing insights into UVR8 dynamics in vivo, in plants growing in both controlled environments and natural daylight. Previous research with plants given acute UV‐B treatments (Rizzini *et al*. 2011) and with purified UVR8 protein (Christie *et al*. 2012; Wu *et al*. 2012) demonstrated that UV‐B induces rapid monomerization of the photoreceptor. Further, it was shown that monomers re‐associate to form dimers following UV‐B exposure, a process that occurs very slowly in vitro but is greatly accelerated in intact cells (Heilmann & Jenkins 2013), in particular by the presence of RUP1 and RUP2 (Heijde & Ulm 2013). The experiments reported here show that UVR8 does not behave like a simple UV‐B switch under photoperiodic growth conditions. If it did, UV‐B exposure at the start of the photoperiod would rapidly induce monomerization and dimers would not reappear until UV‐B exposure ceased at the end of the photoperiod. Such behaviour is not seen; in controlled environment conditions, a photo‐equilibrium is established at the start of the photoperiod and maintained with little change throughout. In daylight, the photo‐equilibrium on a given day is much more variable because of fluctuations in the UV‐B fluence rate and potentially other factors. A possible advantage to the plant of ensuring the presence of UVR8 dimer under constant illumination is that it can respond rapidly to a sudden increase in the level of UV‐B by forming additional monomer, which can initiate acclimation by an appropriate change in gene expression.

An interesting feature of the UVR8 system is that there are parallels with phytochrome photoreceptor dynamics. Both photoreceptors establish a photo‐equilibrium in vivo by cycling between two forms, one of which initiates signalling. However, whereas cycling between Pr and Pfr has been studied extensively (Rockwell *et al*. 2006; Rausenberger *et al*. 2011), little is known about regulation of the UVR8 photo‐equilibrium. The relative rates of UVR8 monomerization and reversion to the dimer appear to be balanced in plants growing in controlled environment conditions so as to maintain the presence of dimer over a range of fluence rates (Fig. 1b). In the present experiments there is evidence of a decrease in %[UVR8^dimer^/UVR8^total^] with increasing UV‐B fluence rate, but the difference is not great considering that the UV‐B fluence rates span an order of magnitude. Hence the dynamics of the system are adjusted to maintain similar levels of dimer and monomer over a wide range of fluence rates. Because *K_p_* will increase with fluence rate, presumably *K_r_* increases in parallel. Thus, in photoperiodic conditions, the regulation of re‐dimerization appears to be at least as important as monomerization.

The RUP proteins are key to regulation of the UVR8 photo‐equilibrium, as demonstrated by the experiments with *rup1*,*rup2* mutant plants. When plants grown without UV‐B are first exposed to UV‐B, monomerization is initiated rapidly (Fig. 2a). However, by the first point in the illumination time course (15 min) it is evident that the *rup1*,*rup2* plants have a higher level of monomer than wild‐type, indicating that RUP‐mediated reversion starts as soon as monomer is formed. Thus, because they are impaired in re‐dimerization, *rup1*,*rup2* plants achieve a much lower %[UVR8^dimer^/UVR8^total^] under photoperiodic conditions (Fig. 2b). Moreover, the relative amount of monomer gradually increases during the photoperiod, indicating that the rate of re‐dimerization fails to match the rate of monomer formation. However, it should be noted that even in *rup1*,*rup2* plants there is still dimer formation, so evidently reversion can occur slowly in the absence of RUPs. Whether proteins other than RUPs can facilitate re‐dimerization, albeit inefficiently, is unknown. The higher level of monomer present in *rup1*,*rup2* plants is consistent with the amplified gene expression and physiological response to UV‐B reported by Gruber *et al*. (2010).

In wild‐type plants, the RUPs evidently have a crucial role in regulating the UVR8 photo‐equilibrium. The proposed increase in *K_r_* to balance *K_p_* at higher UV‐B fluence rates could possibly be achieved by an increase in the amount of RUP proteins as a result of UV‐B stimulated *RUP* expression. It is also possible that UV‐B could regulate RUP activity through some other mechanism, but no information is available in this respect. It is interesting that several factors, including UV‐B, other light qualities and a circadian rhythm, can influence *RUP* expression (Gruber *et al*. 2010; Wang *et al*. 2011), so the regulation of *RUP* expression provides a potential means of modulating the level of UVR8 monomer, and hence UVR8 function, independently of UV‐B.

In daylight, UVR8 photo‐equilibrium does not show a simple relationship with UV‐B fluence rate in individual plants (Fig. 3b–f; Fig. S4), suggesting that it can be influenced by factors apart from fluctuations in UV‐B. Nevertheless, the combined data for 26 sets of plants growing under diverse conditions show that the %[UVR8^dimer^/UVR8^total^] is most strongly correlated with the level of UV‐B over the low fluence range; that is, low ambient fluence rates of UV‐B are most effective in stimulating an increase in monomer. The much smaller effect of fluence rates above 1.5 *μ*mol m^−2^ s^−1^ on %[UVR8^dimer^/UVR8^total^] suggests that a higher rate of monomerization is balanced by a higher rate of re‐dimerization, consistent with the results obtained in controlled environments. The lack of correlation with PAR (other than through the level of UV‐B) is not surprising because there is no indication from any study to date that UVR8 is influenced by light qualities in the 400–700 nm range (Kaiserli & Jenkins 2007; Cloix *et al*. 2012).

It is particularly interesting that the UVR8 photo‐equilibrium can be influenced by temperature. Experiments to monitor the kinetics of re‐dimerization following UV‐B exposure (Fig. 8) indicate that the rate of reversion is reduced at low temperatures, although it is not clear why reversion is slower at 10 °C than 5 °C. The stimulation of reversion at higher temperatures is absent in *rup1*,*rup2* plants, indicating that RUP proteins are required to observe the effect of temperature. However, the basis of the temperature modulation of RUP‐mediated reversion is not known.

Numerous genes are expressed in response to UV‐B exposure and approximately half of these are regulated in a circadian manner over the diurnal period (Takeuchi *et al*. 2014). In many cases maximal expression occurs near the start of the photoperiod to facilitate UV‐protection during sunlight. UVR8 is required for the expression of a large set of UV‐B regulated genes (Brown *et al*. 2005; Favory *et al*. 2009) many of which are subject to circadian regulation; well known examples include *CHS*, *GPO*, *ELIP1*, *ELIP2* and *HYH* (Feher *et al*. 2011; Takeuchi *et al*. 2014). The circadian rhythmicity of UVR8‐regulated gene expression evidently does not match the relatively constant level of UVR8 monomer observed under photoperiodic conditions, indicating that the presence of monomer, although necessary for UVR8 action, is not sufficient to explain the diurnal pattern of gene regulation. Various factors, including the availability of COP1 and its ability to bind to UVR8, which will be influenced by RUP proteins, as well as the abundance and activity of downstream transcriptional regulators will impact on the ability of UVR8 monomer to initiate transcription. Thus, while the formation of UVR8 monomer in response to UV‐B is required for UV‐B regulated expression of many genes, other processes will modulate the pattern of UVR8 regulated gene expression during the diurnal cycle.

## Conclusion

**Figure 9:**
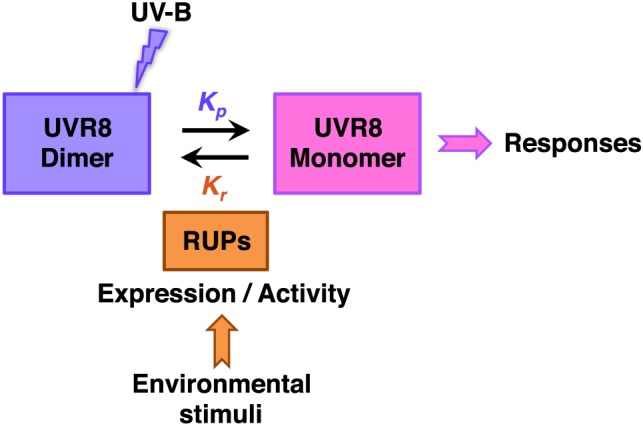
UVR8 dynamics in vivo. UV‐B photoreception by the UVR8 dimer induces monomerization, which leads to the regulation of gene expression and hence a range of responses. In parallel, RUP proteins mediate reversion from monomer to dimer. The rates of monomerization (*K_p_*) and reversion (*K_r_*) are balanced to produce a photo‐equilibrium. The photo‐equilibrium can be influenced by UV‐B and potentially other environmental factors through the regulation of *RUP* gene expression and/or RUP activity.

Figure 9 summarizes the present understanding of UVR8 dynamics in vivo. The photo‐equilibrium ensures that plants maintain a population of dimer over a wide range of UV‐B fuence rates and hence have the capacity to respond rapidly to sudden changes in UV‐B levels to initiate appropriate responses. In addition, the photo‐equilibrium can potentially be influenced by factors other than UV‐B through the regulation of *RUP* gene expression and/or RUP activity, although these aspects of UVR8 regulation are poorly understood. Hence, further research is needed, both to understand how UVR8 controls UV‐B responses in plants growing in photoperiodic conditions and to understand how environmental factors impact on UVR8.

## Supporting information


**Figure S1**. Effect of supplementary UV‐B on leaf area.
**Figure S2**. Quantification of UVR8 dimer/monomer status.
**Figure S3**. Effect of supplementary UV‐B on UVR8 in L*er*.
**Figure S4**. UVR8 dimer/monomer status in plants grown in daylight.

Supporting info item
