# Supplementary material for: Regulation of UVR8 photoreceptor dimer/monomer photo‐equilibrium in Arabidopsis plants grown under photoperiodic conditions
Source: Plant Cell Environ. 2016 May 6;39(8):1706–14. doi: 10.1111/pce.12724 (PMC5103188; doi:10.1111/pce.12724)
Supplement: Supplementary file 1 — Supporting info item [file PCE-39-1706-s001.pdf]

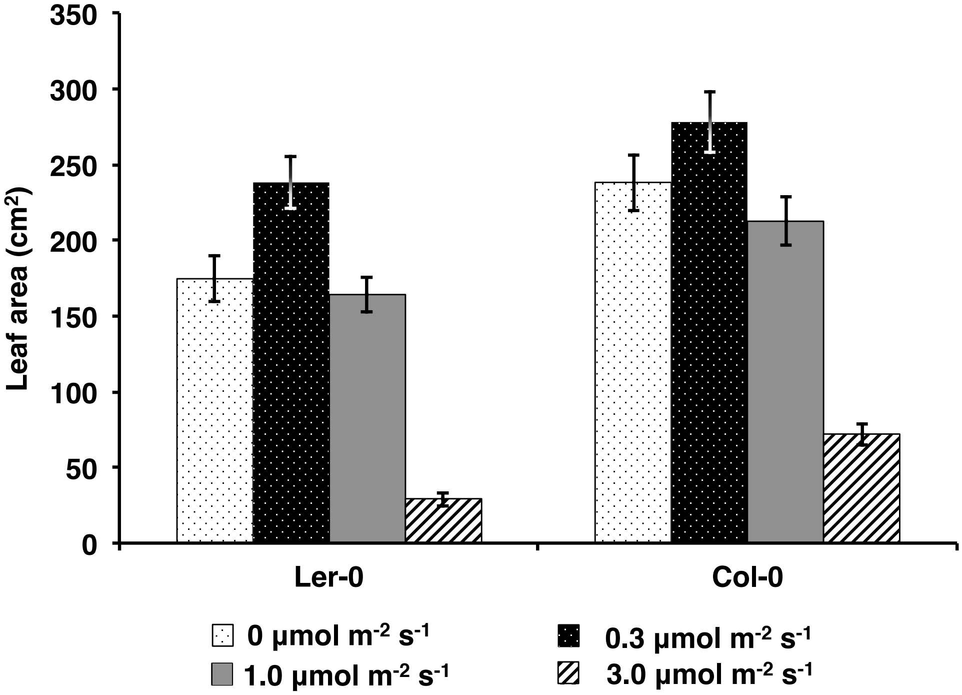

Figure S1. Effect of supplementary UV-B on leaf area.
Total leaf area was measured for Col-0 and L*er* plants grown in controlled environment cabinets for 3 weeks under 120 μmol m^{-2} s^{-1} white light with either no UV-B or supplementary UV-B at 0.1, 1.0 or 3.0 μmol m^{-2} s^{-1}. Data are $\pm$ S.E. (n=6).

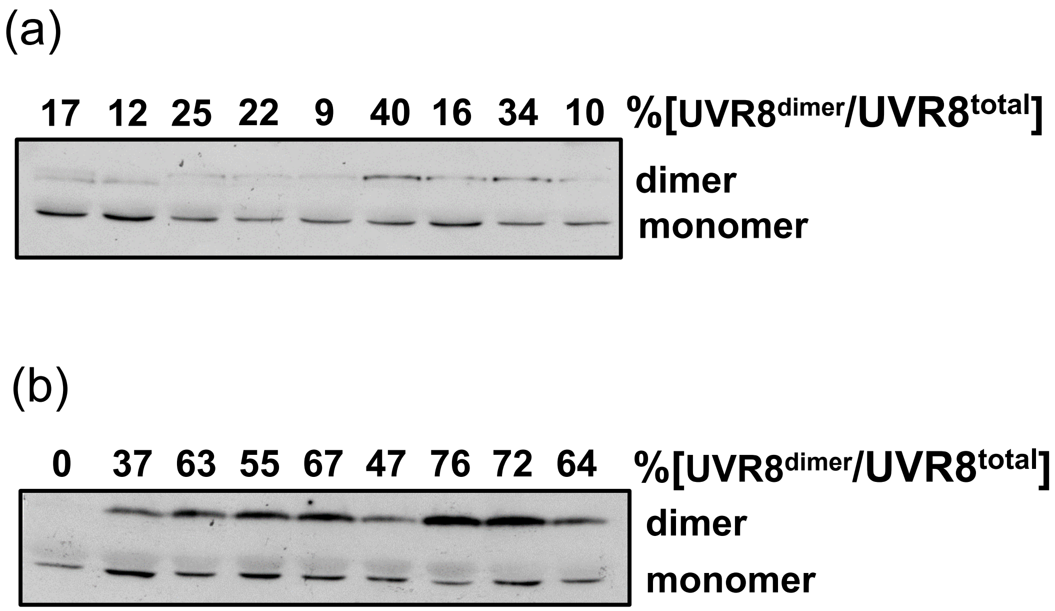

Figure S2. Quantification of UVR8 dimer/monomer status.
Two western blots are shown as examples of the quantification of UVR8 dimer/monomer data. The blots are for samples obtained in experiments with plants grown in daylight (upper blot: Fig. S4 panel H; lower blot: Fig. S4 panel P). Quantification was undertaken by chemiluminescent imaging following immunodetection, as described in Materials and Methods. The values above the lanes of the blot are the quantified %[$UVR8^{dimer}$/$UVR8^{total}$] for each protein sample.

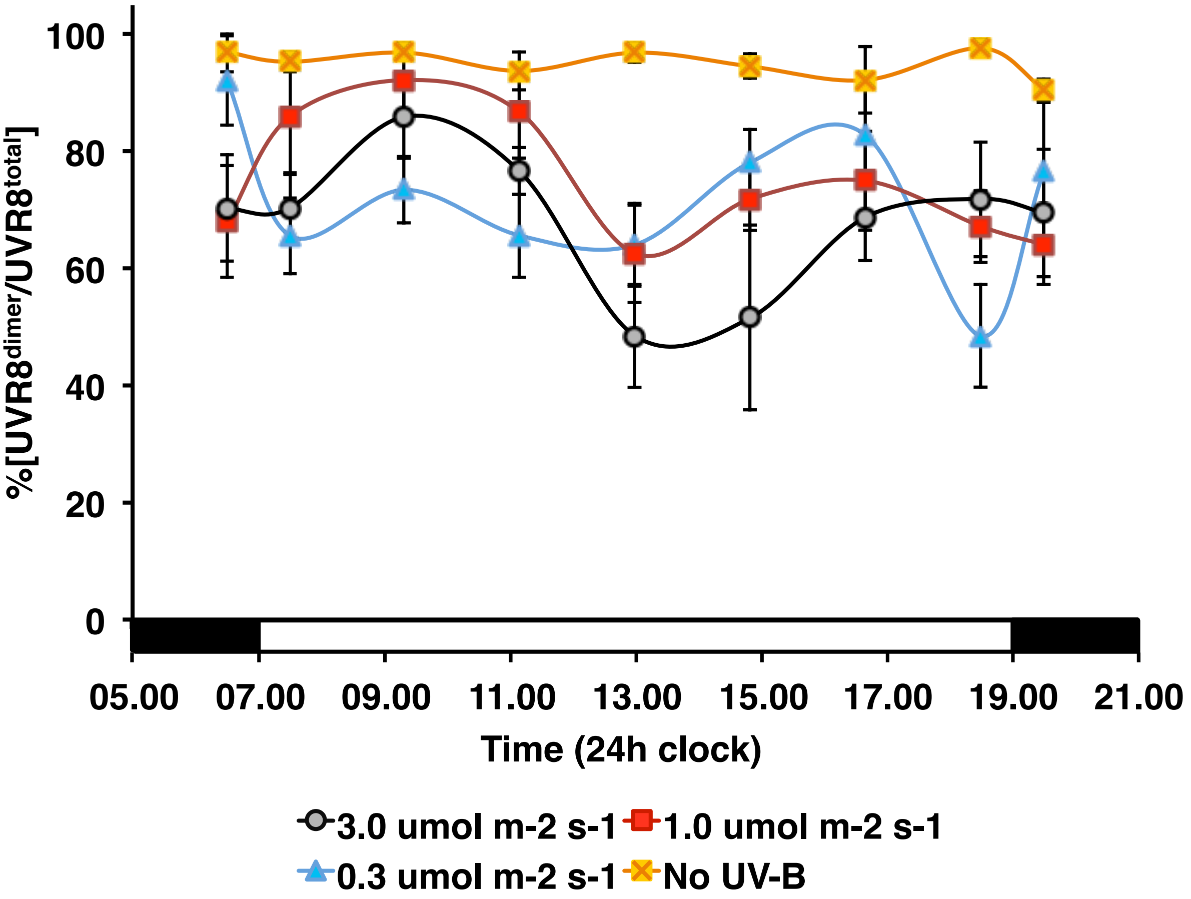

Figure S3. Effect of supplementary UV-B on the UVR8 photoequilibrium in L*er* plants.
Plants were grown and analysed as in Fig. 1b.

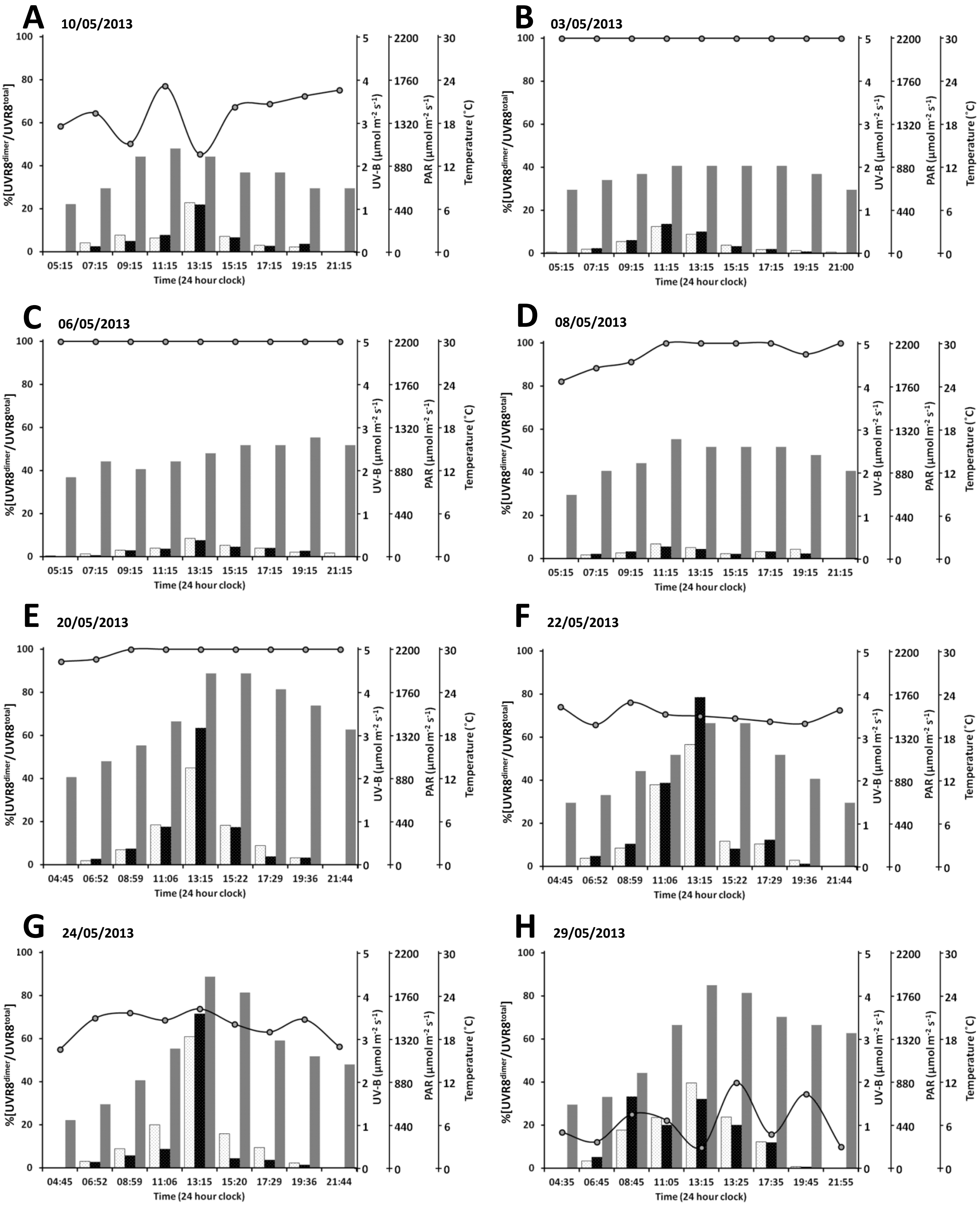

A
10/05/2013
B
03/05/2013
C
06/05/2013
D
08/05/2013
E
20/05/2013
F
22/05/2013
G
24/05/2013
H
29/05/2013
%[UVR8^dimer/UVR8^total]
UV-B (µmol m^-2 s^-1)
PAR (µmol m^-2 s^-1)
Temperature (°C)
Time (24 hour clock)
0 20 40 60 80 100
0 1 2 3 4 5
0 440 880 1320 1760 2200
0 6 12 18 24 30
05:15 07:15 09:15 11:15 13:15 15:15 17:15 19:15 21:15
05:15 07:15 09:15 11:15 13:15 15:15 17:15 19:15 21:00
04:45 06:52 08:59 11:06 13:15 15:22 17:29 19:36 21:44
04:45 06:52 08:59 11:06 13:15 15:20 17:29 19:36 21:44
04:35 06:45 08:45 11:05 13:15 13:25 17:35 19:45 21:55

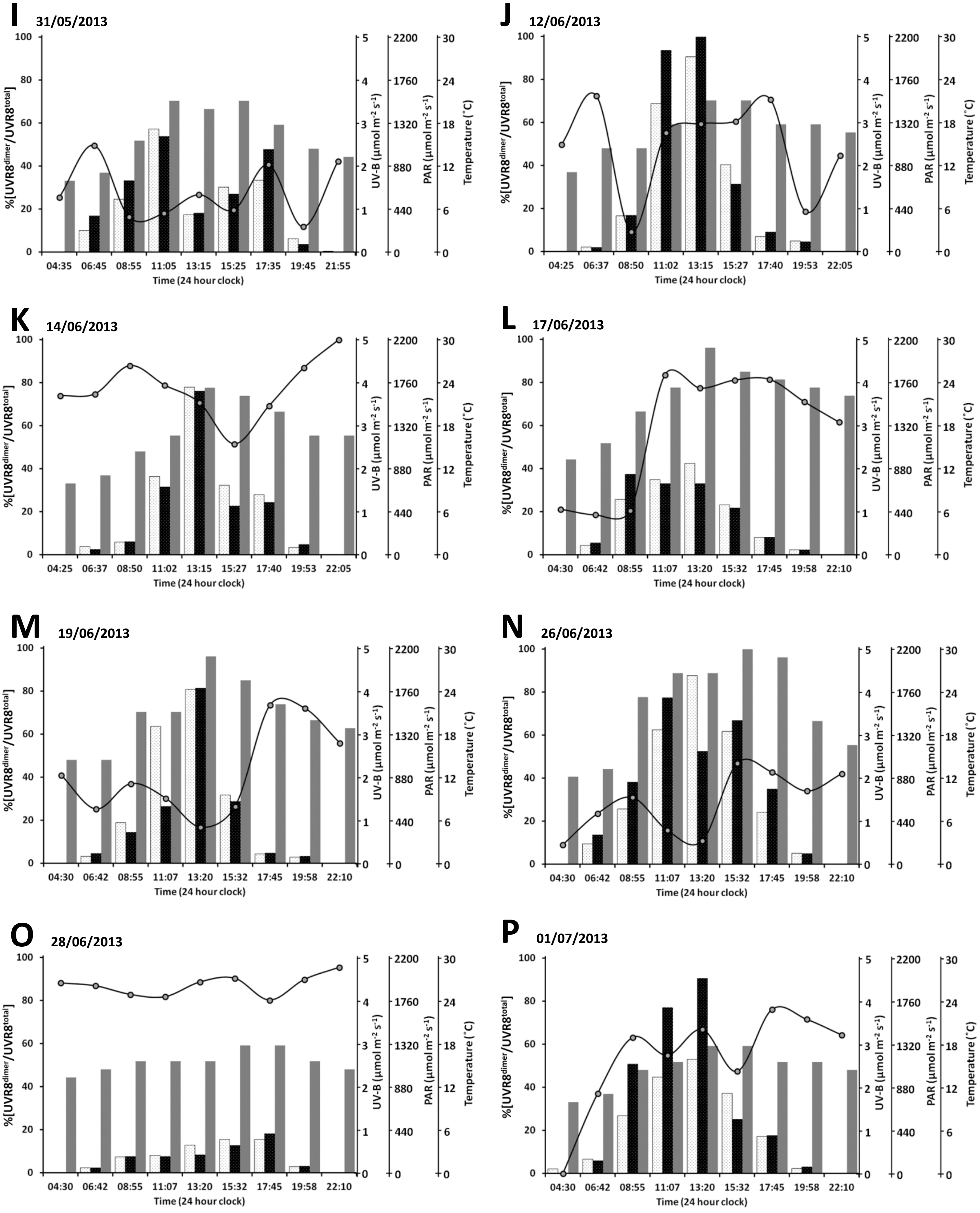
I 31/05/2013
J 12/06/2013
K 14/06/2013
L 17/06/2013
M 19/06/2013
N 26/06/2013
O 28/06/2013
P 01/07/2013
%[UVR8dimer/UVR8total]
UV-B (μmol m-2 s-1)
PAR (μmol m-2 s-1)
Temperature (°C)
Time (24 hour clock)

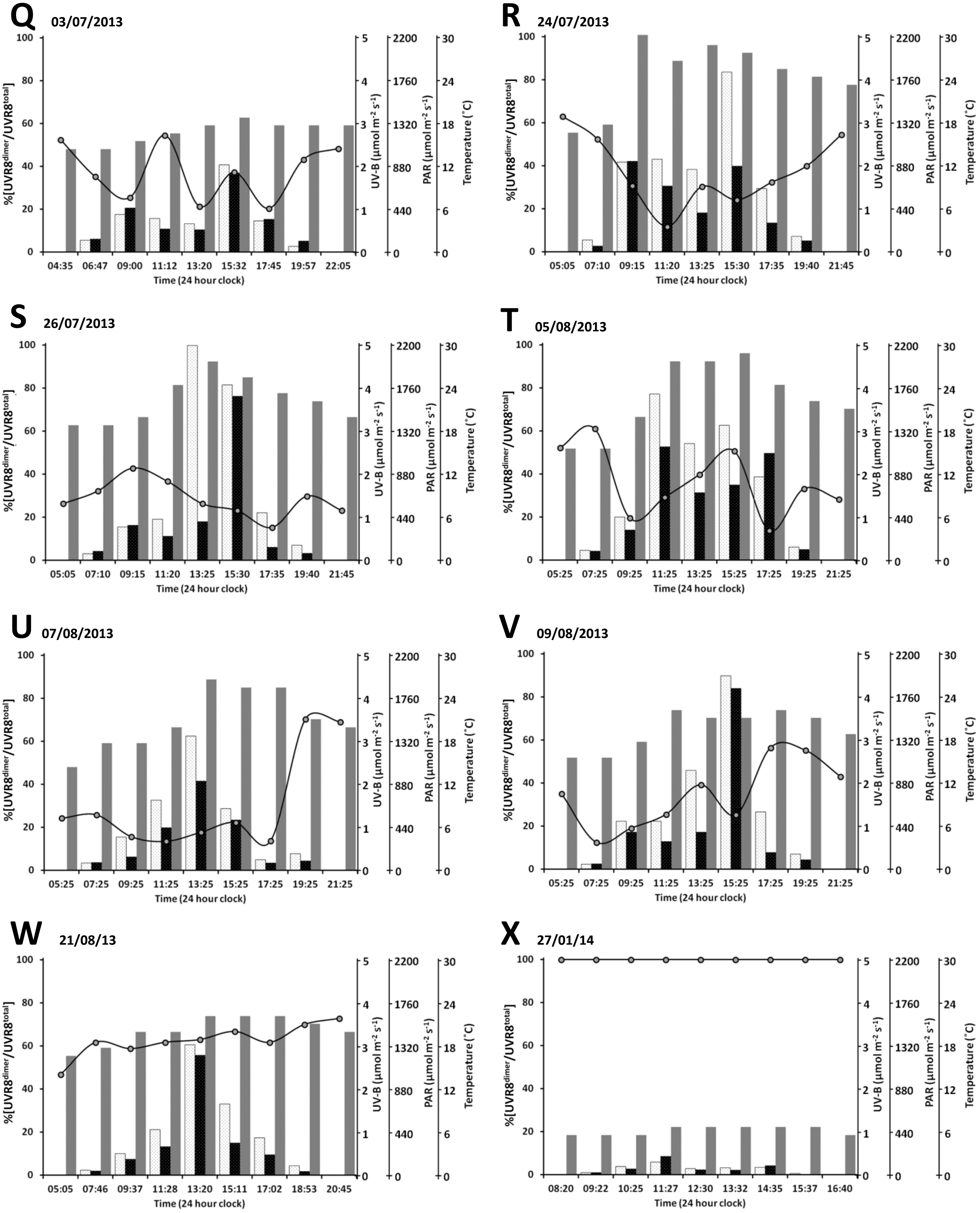
Q 03/07/2013
R 24/07/2013
S 26/07/2013
T 05/08/2013
U 07/08/2013
V 09/08/2013
W 21/08/13
X 27/01/14
%[UVR8^dimer/UVR8^total]
UV-B (µmol m^-2 s^-1)
PAR (µmol m^-2 s^-1)
Temperature (°C)
Time (24 hour clock)
0 20 40 60 80 100
0 1 2 3 4 5
0 440 880 1320 1760 2200
0 6 12 18 24 30
04:35 06:47 09:00 11:12 13:20 15:32 17:45 19:57 22:05
05:05 07:10 09:15 11:20 13:25 15:30 17:35 19:40 21:45
05:25 07:25 09:25 11:25 13:25 15:25 17:25 19:25 21:25
05:05 07:46 09:37 11:28 13:20 15:11 17:02 18:53 20:45
08:20 09:22 10:25 11:27 12:30 13:32 14:35 15:37 16:40

Y 31/01/14

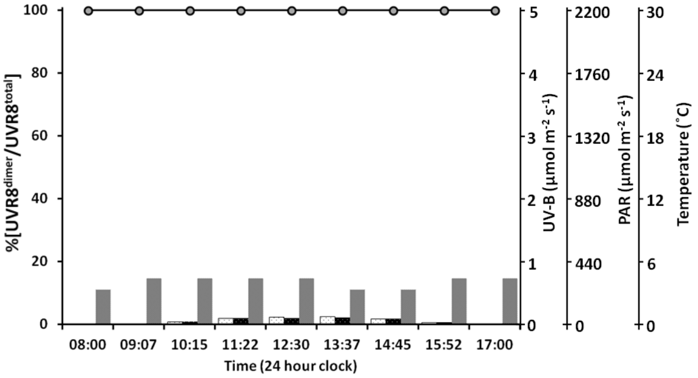

Z 03/02/14

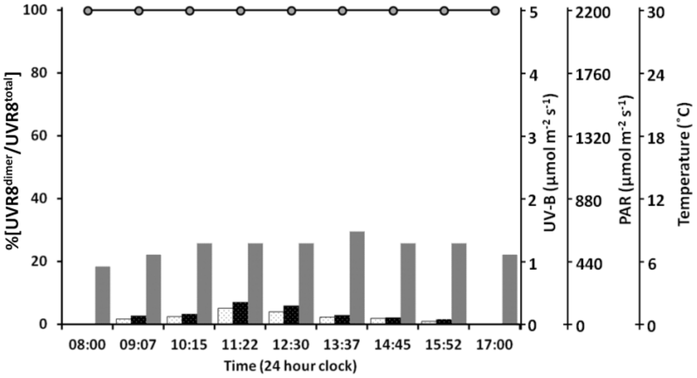

Figure S4. UVR8 dimer/monomer status in plants grown in daylight. Measurements were recorded on 26 days in Glasgow UK (A to Z; dates indicated). Plants were grown initially for 7 days in 120 μmol m^{-2} s^{-1} white light in a controlled environment and then transferred to daylight for 2 weeks, after which measurements were made throughout a single day. The first and last time points are approximately 30 mins before dawn and after dusk respectively. The 5th time point is at solar noon. Data are shown for %[$UVR8^{dimer}$/$UVR8^{total}$] in leaf samples, UV-B, PAR and temperature at each time point. The data in E, H, K, U and W are also shown in Fig. 3 but are included here for completeness and comparison with the other data.
